# Patient-Generated Data Analytics of Health Behaviors of People Living With Type 2 Diabetes: Scoping Review

**DOI:** 10.2196/29027

**Published:** 2021-12-20

**Authors:** Meghan S Nagpal, Antonia Barbaric, Diana Sherifali, Plinio P Morita, Joseph A Cafazzo

**Affiliations:** 1 Institute of Health Policy, Management and Evaluation University of Toronto Toronto, ON Canada; 2 Centre for Global eHealth Innovation Techna Institute University Health Network Toronto, ON Canada; 3 Institute of Biomedical Engineering University of Toronto Toronto, ON Canada; 4 School of Nursing McMaster University Hamilton, ON Canada; 5 School of Public Health and Health Systems University of Waterloo Waterloo, ON Canada; 6 Department of Computer Science University of Toronto Toronto, ON Canada

**Keywords:** type 2 diabetes, obesity management, health behavior, machine learning, artificial intelligence, big data, data science, patient-generated health data, mobile phone

## Abstract

**Background:**

Complications due to type 2 diabetes (T2D) can be mitigated through proper self-management that can positively change health behaviors. Technological tools are available to help people living with, or at risk of developing, T2D to manage their condition, and such tools provide a large repository of patient-generated health data (PGHD). Analytics can provide insights into the health behaviors of people living with T2D.

**Objective:**

The aim of this review is to investigate what can be learned about the health behaviors of those living with, or at risk of developing, T2D through analytics from PGHD.

**Methods:**

A scoping review using the Arksey and O’Malley framework was conducted in which a comprehensive search of the literature was conducted by 2 reviewers. In all, 3 electronic databases (PubMed, IEEE Xplore, and ACM Digital Library) were searched using keywords associated with diabetes, behaviors, and analytics. Several rounds of screening using predetermined inclusion and exclusion criteria were conducted, after which studies were selected. Critical examination took place through a descriptive-analytical narrative method, and data extracted from the studies were classified into thematic categories. These categories reflect the findings of this study as per our objective.

**Results:**

We identified 43 studies that met the inclusion criteria for this review. Although 70% (30/43) of the studies examined PGHD independently, 30% (13/43) combined PGHD with other data sources. Most of these studies used machine learning algorithms to perform their analysis. The themes identified through this review include predicting diabetes or obesity, deriving factors that contribute to diabetes or obesity, obtaining insights from social media or web-based forums, predicting glycemia, improving adherence and outcomes, analyzing sedentary behaviors, deriving behavior patterns, discovering clinical correlations from behaviors, and developing design principles.

**Conclusions:**

The increased volume and availability of PGHD have the potential to derive analytical insights into the health behaviors of people living with T2D. From the literature, we determined that analytics can predict outcomes and identify granular behavior patterns from PGHD. This review determined the broad range of insights that can be examined through PGHD, which constitutes a unique source of data for these applications that would not be possible through the use of other data sources.

## Introduction

### Background

Diabetes is a serious metabolic condition in which the body experiences elevated blood glucose levels that can result in serious complications such as cardiovascular disease, kidney disease, stroke, eye disease, foot ulcers, nerve damage, and amputation. The World Health Organization has stated that high blood glucose levels are the third leading cause of premature mortality [1]. As of 2015, it is estimated that globally 415 million adults are living with diabetes, with 3.4 million in Canada; the latter number is expected to rise to 5 million, or 12.1% of the Canadian population, by 2025 [2]. Type 2 diabetes (T2D) is characterized by the body’s resistance or insufficient production of insulin. Research suggests that the risks of further complications for people living with T2D can be mitigated through proper self-management [3]. The treatment protocol for proper management of T2D includes glycemic control, weight management, adequate nutrition, regular physical activity, sedentary behavior reduction, and medication adherence [4].

Technology-enabled tools may facilitate behavior change in people living with, or at risk of developing, T2D and help to manage their condition by delivering tailored feedback. Mobile health (mHealth) options through smartphones, mobile apps, wearable sensors, smartwatches, and additional devices that include Bluetooth-enabled blood glucose meters (BGMs), bodyweight scales, and commercial blood pressure monitors provide low-cost and accessible tools for self-management of diabetes [5]. These interventions have resulted in reductions of glycated hemoglobin of between 0.5% and 0.8% and an average weight loss of 2.4 kg [6,7]. Users of mHealth options for managing T2D reported higher satisfaction, better quality of life, self-efficacy, and potential for increased treatment adherence [7]. The emergence of web and mobile apps and internet-enabled sensory devices has resulted in the creation of a large repository of patient-generated health data (PGHD) [8,9]; in the context of health care, the sources of these data include sensors, social media posts, blogs, and smartphone activity [10]. In contrast to sources generated by clinicians, such as electronic medical records (EMRs), PGHD can provide a firsthand view of the behaviors of people living with, or at risk of developing, T2D because the data are generated directly from the consumer as well. These sources could include data from mHealth apps such as smartphone apps, from Bluetooth-enabled medical devices such as BGMs, or from social media platforms such as Twitter.

### Advanced Analytical Techniques

Large volumes of data, or *big data*, can provide information through analytics, which is defined as the process of systematically using data to derive insights by using applied analytical disciplines to facilitate decision-making [11]. Traditionally, analytical insights were derived from statistical models. However, with the emergence of big data, machine learning (ML) and artificial intelligence (AI) have come to the fore as advanced analytical techniques in which computers automatically extract patterns from data [9,12]. Raghupathi and Raghupathi [10] express that “explosions” of data volumes from the aforementioned data sources have the ability to “improve care, save lives, and lower costs” [10], with PGHD from diabetes management tools being no exception. In particular, the benefit of PGHD from diabetes management tools is that there is opportunity to use analytics to derive insights into the health behaviors of people living with T2D because these data are generated directly from the consumer, with greater frequency and context, and not solely from the perspective of an infrequent observer such as a clinician. Health behaviors include techniques for self-management of T2D that encompass proper glycemic control, eating a healthy diet, increasing physical activity, reducing sedentary time, and taking prescribed medications. Analytics can describe current health behaviors of people living with T2D and make predictions about health outcomes and prescribe treatment recommendations based on these behaviors. The aim of this review is to consolidate the current literature on what has already been learned from analytics from PGHD of the health behaviors of people living with, or at risk of developing, T2D.

## Methods

### Scoping Review Framework

This review followed the scoping review framework of Arksey and O’Malley [13] using the following steps: (1) identifying the research question, (2) identifying relevant studies, (3) study selection, (4) charting the data, and (5) summarizing and reporting the results [14]. This scoping review methodology was chosen to identify the scope of research surrounding analytics of health behaviors gathered through PGHD from people living with, or at risk of developing T2D, and to map concepts obtained from the literature [15].

### Research Question

Although data from EMRs or administrative sources can provide insights into clinical outcomes, they are obtained from the perspective of health care providers or administrators. Data that are obtained directly from the consumer can provide descriptive analytical insights into their health behaviors and predictive and prescriptive insights from these behaviors. This led to developing the following research question:

What is known in the current literature about analytical insights about health behaviors that have been derived from PGHD from people living with, or at risk of developing, T2D?

### Search Strategy

Searches were conducted in July 2020 through 3 databases, PubMed, IEEE Xplore, and ACM Digital Library, using the search terms diabetes, behaviors, and analytics. Related keywords were refined as described in Textbox 1*.* Search terms were limited to *Title* and *Abstract* for studies in PubMed and *Abstract* for studies in IEEE Xplore and ACM Digital Library because there is no option to search for *Title* and *Abstract* in these 2 databases. The years of publication were limited to 2010-2020. Using the keywords identified, 2 reviewers (MSN and AB) conducted searches through the 3 databases and identified relevant studies using the inclusion and exclusion criteria. For review articles and studies included in the data charting phase, reference lists were scanned, and additional studies that were not found through the initial search were extracted. Studies that were not complete, those whose full text was not available, and those that were not published in English were not included.

**Search terms for the scoping review.**

**Search terms**

- *Diabetes* AND (Behav* OR Coach*) AND (*Artificial Intelligence* OR *Big Data* OR *Machine Learning* OR *Analytics* OR *Decision Support* OR Knowledge Engineer* OR Intelligent Retriev* OR Expert System* OR Business Intell*)

### Study Selection

Following the Arksey and O’Malley framework [13], articles were reviewed in 3 iterations. In the first iteration, abstracts were scanned and selected using the eligibility criteria (see *Inclusion Criteria* and *Exclusion Criteria* sections). In the second iteration, the full text was scanned using the same eligibility criteria, after which articles were selected. In the final iteration, data were extracted and charted, and studies were excluded if they did not meet the eligibility criteria.

For the purposes of this review, PGHD are defined as data that were generated directly from the patient through devices that are already available for consumer use. These would include data inputted directly by patients through mobile apps; data collected passively through wearable devices such as smartwatches or accelerometers as well as data from BGMs, continuous glucose monitors (CGMs), or insulin pumps; data obtained from social media platforms such as Reddit or Twitter; or data obtained from patient surveys or questionnaires. Although some of these studies integrated PGHD with other data sources such as EMRs, administrative health data, or census data, all included studies must have included at least one source of PGHD.

Eligibility was determined using the inclusion and exclusion criteria listed below. For an article to be included, it must have met all inclusion criteria and not have met any exclusion criteria.

### Inclusion Criteria

The inclusion criteria are:

1. Primary intervention driven by an analytical method AND
2. Target population includes people living with, or at risk of developing, T2D (eg, people living with obesity) AND
3. Study objective primarily focuses on health behaviors AND
4. Study must include at least one source of PGHD

### Exclusion Criteria

The exclusion criteria are:

1. Technologies that are not generally available at the consumer level (ie, prototype or investigational devices) OR
2. Theoretical models that have not been applied on actual data OR
3. Studies that have not been completed OR
4. Studies not published in English OR
5. Review articles (reference lists of review articles were scanned and articles were directly extracted) OR
6. Commentary and gray literature (ie, letters, commentary, editorials, blogs, and news articles)

### Charting and Extracting Data

Articles meeting the inclusion criteria were examined and critically evaluated using the descriptive analytical method outlined by Arksey and O’Malley [13]. MSN created data parameters to guide extraction, and these parameters included year of publication, study goals, source of study data, study type, analytical method (ie, algorithms used on data), analytics type (ie, descriptive, predictive, or prescriptive), and main findings. Study goals were directly extracted and quoted from the article when available, and the remaining data parameters were interpreted through analysis from examining the article.

### Summarizing and Reporting Results

The descriptive data were examined manually by MSN, and themes were identified and given numerical codes. These themes were categorized and organized into thematic groups to summarize the studies by their main findings. Doing this enabled us to present a narrative to answer our research question.

## Results

### Overview

**Figure 1:**
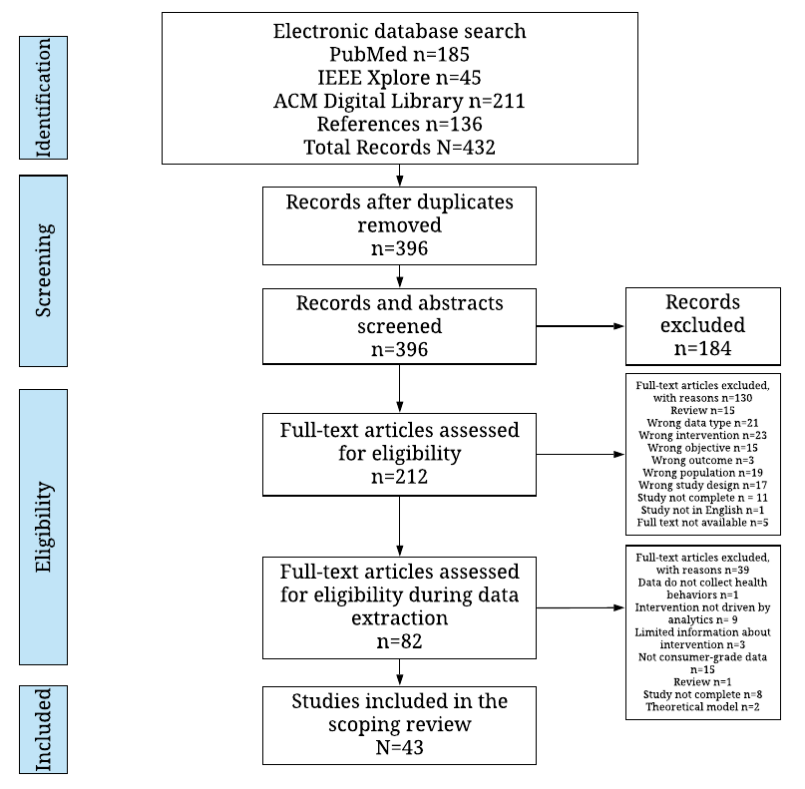
PRISMA (Preferred Reporting Items for Systematic Reviews and Meta-Analyses) flowchart: systematic study selection.

A total of 432 articles were identified from 3 databases and reference list searches. Of these 432 articles, 36 (8.3%) were duplicates and were subsequently removed. The abstracts and full texts of the remaining 396 articles were screened by MSN and AB, and 83 (20.9%) were included for data extraction. Finally, after close examination from data extraction by MSN, of the 83 articles, 43 (52%) were included as part of this scoping review. Figure 1 summarizes the process.

### Study Characteristics

A total of 43 studies published from 2012 to 2020 were included in this review. With respect to study design, of the 43 studies, 18 (42%) incorporated a cross-sectional study, 12 (28%) incorporated an analytical framework or algorithm, 10 (23%) incorporated a comparative study, 2 (5%) incorporated a randomized controlled trial, 1 (2%) was a cohort study, 1 (2%) was a longitudinal study, and 1 (2%) was a mixed methods study.

Of the 43 studies, 30 (70%) examined only PGHD, whereas 13 (30%) combined PGHD with another source. Sources of PGHD include surveys and interviews (14/43, 33%); activity sensors (12/43, 28%); social media and forums (9/43, 21%); mHealth apps (6/43, 14%); CGMs, BGMs, and insulin pumps (3/43, 7%); self-reported data (3/43, 7%); open data sets (2/43, 5%); and web applications (1/43, 2%). The other sources of data that were not consumer generated included demographic data (5/43, 12%), external knowledge or external databases (4/43, 9%), EMRs and clinical data (3/43, 7%), laboratory data (2/43, 5%), and administrative data (1/43, 2%).

Of the analytic types examined through the data sources, of the 43 studies, 19 (44%) used descriptive analytics, 24 (56%) used predictive analytics, and 5 (12%) used prescriptive analytics. Most of the studies used AI or ML algorithms for data analysis; 93% (40/43) used an AI-based algorithm alone or in combination with a traditional statistical method, whereas only 7% (3/43) used traditional statistical methods.

### Findings From Thematic Analysis

The findings from this review suggest a broad range of themes pertaining to analytical insights through PGHD from people living with, or at risk of developing, T2D and are summarized in Multimedia Appendix 1 [16-58]. A total of 9 themes are classified into 4 categories as follows:

1. Forecasting clinical correlations and outcomes
  Predicting diabetes and obesity (10/43, 23%)
  Predicting glycemia (6/43, 14%)
  Discovering clinical correlations from behaviors (3/43, 7%)
2. Understanding patient behaviors
  Deriving factors that contribute to diabetes and obesity (8/43, 19%)
  Obtaining insights from social media and web-based forums (7/43, 16%)
  Analyzing sedentary behaviors (5/43, 12%)
  Deriving behavior patterns (4/43, 9%)
3. Facilitating treatment Interventions
  Improving adherence and outcomes (5/43, 12%)
4. Improving technology
  Developing design principles (3/43, 7%)

### Forecasting Clinical Outcomes

#### Overview

Analytics have the ability to forecast patient outcomes using a combination of descriptive and predictive analytics. Predictive analytics can predict adverse events before they occur, making it possible to prevent them from occurring, and descriptive analytics can describe current patterns that, in turn, can forecast clinical likelihoods. This review found that the prevalent themes in this category are predicting diabetes and obesity, predicting glycemia, and discovering clinical correlations from behaviors.

#### Predicting Diabetes and Obesity

The most prevalent theme in this review was using PGHD to predict the likelihood of diabetes or obesity through PGHD [16-25] from health behaviors. Considering that 80%-90% of the people living with T2D are overweight or obese [59,60], the risk of obesity was considered as a precursor condition to developing T2D.

Of these 10 studies, 9 (90%) used survey or questionnaire data to make these predictions; of these 9 studies, 7 (78%) were comparative studies in which different ML algorithms were compared for accuracy in predicting obesity and diabetes. Meng et al [16] and Abdullah et al [19] found that performing decision tree algorithms on survey and questionnaire data was the most successful in predicting childhood obesity with an accuracy of 82.63% and diabetes with an accuracy of 77.87%, respectively. Choi et al [17] and Han et al [18] found support vector machine (SVM) models on data from national health and nutrition surveys conducted in Korea and China to be the most accurate at predicting diabetes risk, with an area under the receiver operating characteristic curve value of 0.731 and an accuracy of 89.6%, respectively. However, the model presented by Han et al [18] integrates SVM with random forest (RF) because they had more success with the integrated approach than by using SVM alone [18]. Other studies suggested that logical regression analysis, naive Bayes, gradient boosting, RF with AdaBoost, and recurrent neural network were accurate in predicting obesity or diabetes through survey data, EMR data, and activity data from wearables, with accuracies ranging from 72% to 99% [20,22-25].

#### Predicting Glycemia

From the studies identified, blood glucose levels could be predicted through information about food intake, exercise, medications, insulin, sleep, and blood glucose readings entered through web applications, mobile apps, smartphone activity sensors, or BGMs [26-29]. These studies showed promising results, with Hidalgo et al [26] predicting hypoglycemia with 79%-100% accuracy and hyperglycemia with 74%-97% accuracy, Gu et al [27] predicting blood glucose levels with accuracy of 84.14%, and Faruqui et al [29] accurately predicting next-day blood glucose levels with a Clarke Error Grid and a range of −10% to 10% of actual values. Heuschkel and Kauschke [30] used data from CGMs and insulin pumps as well as smartphone movements and heartrate sensors to predict glycemia and found that their algorithm performed slightly better than commercial insulin pumps (mean absolute error 8.74 for the model during 15-minute intervals vs mean absolute error of 10.10 with the insulin pump) [30]. However, this model was still unable to detect crisis situations. Machado [28], however, developed a framework to detect glycemic crisis situations from mobile app data using rule-based logic. Finally, Namayanja and Janeja [31] examined the University of California, Irvine, Diabetes Data Set to derive clusters of behavior patterns correlating to insulin dosage and blood glucose levels to determine at which specific time periods people living with T2D had more imminent needs.

#### Discovering Clinical Correlations From Behaviors

Examining PGHD provides the opportunity to examine clinical correlations from the health behaviors of people living with T2D. In the study by Chen et al [32], it was found that strong social connections increased physical activity, with Fitbit data from participants seeing an increase of average daily step count from 6332 to 6631 after the establishment of a strong social connection. This study demonstrated through analysis of PGHD from patients living with diabetes that a positive correlation exists between strong social connections and physical activity level. Another study that used Fitbit data was by Weatherall et al [33], who used Fitbit data combined with patient-reported outcomes to examine the correlation of patient outcomes with physical activity and sleep. They found a positive correlation of better patient outcomes with increased physical activity and sleep that was captured by Fitbit data. Finally, Sarda et al [34] examined depressive characteristics of people living with T2D by examining smartphone activity. They found among a sample of people living with diabetes that lower smartphone activity and decreased social contacts correlated with increased symptoms of depression. All these studies demonstrate that PGHD offer a unique opportunity to uncover correlations between health behaviors and clinical outcomes by analyzing passive activity through device use; both Chen et al [32] and Weatherall et al [33] used passive data collected through Fitbit to discover their findings, whereas Sarda et al [34] used data collected through passive smartphone activity.

### Understanding Patient Behaviors

#### Overview

With the ability to collect large volumes of data both actively and passively, analytics provide clinicians with a more detailed account of the health behaviors of patients. Clinicians can then understand the behavior patterns of patients and the factors that affect their clinical outcomes.

#### Deriving Factors That Contribute to Diabetes and Obesity

This review found that through PGHD it was possible to derive the factors responsible for obesity or diabetes [21,23,25,35-39]. Height, weight, BMI, and weight loss were anthropometric measurements that correlated to the incidence of diabetes and obesity [21,23,25,37,39], and age was a demographic variable that was also predictive of diabetes and obesity [23,37]. Diet and sleep were lifestyle behaviors contributing to diabetes and obesity [23,35,37,38], with Xie et al [38] suggesting that sleeping for 9 hours or more per day increases the risk of developing diabetes. Data sources from these studies included not only questionnaires, but also more passive sources, which included CGMs and sensors as well as social media discussions.

#### Obtaining Insights From Social Media and Web-Based Forums

Social media and web-based forums provide platforms for people living with T2D to discuss their condition and related information among their peers [40]. The themes that emerged through web-based discussions include diet, food, symptoms, research, recipes, and news [35,37,40,41]. All studies under this theme used social media data as their data source. Abbar et al [35] and Griffis [41] found that tweets posted on Twitter about unhealthy foods correlate to geographical areas with higher incidences of obesity and diabetes. Sentiment analysis of social media posts suggested a negative correlation of positive emotions and blood glucose levels for people living with diabetes [42] and a correlation of negative emotions to higher weight loss [39]. Finally, social media connections have been shown to influence behaviors that lead to obesity; Wilder et al [43] created an algorithm in which participants updated their behavior under the influence of the people around them, averting 230 cases of obesity.

#### Analyzing of Sedentary Behaviors

Activity sensors provided a source of PGHD that could be analyzed to determine sedentary behaviors (time spent by the user being stationary while awake). Reducing sedentary behaviors is considered to be a positive health behavior in the treatment of T2D. Li et al [44] found that the rotating forest algorithm was the most successful at predicting sedentary behaviors through sensors, with an accuracy of 73%. He and Agu [45] found that people’s future sedentary behaviors can be predicted by historic sedentary behaviors in previous 6-hour windows, with patterns being repeated daily and weekly, and subsequently, in a later study, they found that the rhythms of sedentary behavior tend to be cyclical, as opposed to linear [46]. Xiao et al [47] developed a framework using the demographic feature hidden Markov model to predict the trajectory of latent states using synthetic and sensor data. Early prediction of sedentary behaviors can potentially alert the user to move about and reduce stationary time.

Activity sensors that detect sedentary behaviors can also find clinical correlations from stationary patterns. Chang et al [48] found that longer sitting time was associated deleteriously with higher fasting insulin and triglyceride concentrations, insulin resistance, and increased BMI, and waist circumference among female participants, with the correlation between mean sitting bout duration and fasting blood glucose concentration being significantly stronger among Hispanic women than among non-Hispanic women.

All these studies used data from some form of activity sensor and demonstrated the unique perspective provided by PGHD through activity sensors: the ability to monitor the daily physical movements of users and provide an accurate measurement of sedentary behaviors and subsequently reduce them, if necessary, as a means of treating T2D.

#### Deriving Behavior Patterns

Large volumes of PGHD can help to detect different combinations of health behavior patterns of people living with T2D, which may not necessarily be captured through other data sources. Exploring behavior patterns can potentially unveil correlations among different health behaviors and can better advise users to make necessary changes. In the study by Machado et al [28], a mobile app was developed to allow users to record their meals, exercise sessions, and blood glucose levels, and a rule-based system would advise users about crisis situations. Namayanja and Janeja [31] captured granular behavior patterns correlating to blood glucose level and insulin dosage through k-means clustering, which was more accurate than statistical analysis. Tirunagari et al [49] further captured behavior patterns using self-organizing maps and found that those who took correct insulin dosages took them at the right time, those who ate on time ate the correct portions, and those who regularly checked their blood glucose levels carried snacks or took correct insulin dosages. Finally, Seixas et al [50] examined behavior patterns to investigate diabetes prevalence by race. They found that physical activity with low stress, adequate sleep, and average body weight reduced the diabetes risk among Black people. These studies used some form of survey and questionnaire data to derive these patterns. Analytics from PGHD have the ability to recognize patterns of health behaviors and infer correlations as a result of these patterns.

### Facilitating Treatment Interventions

#### Overview

Technological tools to manage T2D have the ability to help people manage their treatment by improving adherence to behavior changes, alerting users about predicted adverse events, and prescribing recommendations for behavior change.

#### Improving Adherence and Outcomes

Analytics from PGHD can be used to improve adherence to treatment as well as overall outcomes for people living with T2D [28,51-54]. Prescriptive interventions that advise users and personalize messages have been shown to improve adherence to treatment interventions [51,54]. In Feller et al [54], visual analytics and hierarchal clustering determined that users assigned to use a web- and mobile-based diabetes app displayed 50% more use than those who used static logbooks. Of the 43 studies, 2 (5%) were frameworks with intentions to improve user outcomes: Nag et al [52] devised personalized meal recommendations using nutritional and restaurant databases, with findings validated by a dietitian, and Machado et al [28] analyzed behavior patterns in regard to nutrition management, exercise, and glycemic control and used rule-based logic to advise users about potential crisis situations. All studies used a form of self-reported data through an app through nutrition logs, blood glucose readings, and activity sensors.

### Improving Technology

#### Overview

As technologies are used and tested, newer technologies have the opportunity to improve on previous generations by analyzing feedback and results from users. In the next section, we discuss studies that used PGHD to create frameworks for new technologies.

#### Developing Design Principles

The development of frameworks using PGHD and close examinations of user feedback have given insight into general design principles of creating a technical intervention to help people living with T2D to manage their condition. Al-Ramahi et al [55] examined user reviews of diabetes management mobile apps on the iTunes store and determined that the most important design principles were “effort expectancy,” “self-monitoring,” “informative presentation,” “communication with doctors,” and “integration with information systems.” Other critical design principles were “integration with medical devices,” “customization,” and “technical support” [55]. Fong et al [56] created a framework for a clinical decision support system for diabetes therapy and found that a system needs to (1) be able to handle live streams, (2) have a short time delay, and (3) have accurate and consistent performance. Finally, Albers et al [57] created a system that generated personalized blood glucose–level forecasts that had the following attributes: (1) estimated data in real time according to metrics; (2) forecast in line with the opinions of certified diabetes educators; (3) personalized the model to the individual; (4) integrated with model selection machinery and chose the best model; (5) performed well, given realistic data; (6) produced accurate output, and (7) averaged in real time to produce accurate forecasts.

## Discussion

### Principal Findings

The goal of this review is to understand what we can learn from analytics from PGHD about the health behaviors of people living with, or at risk of developing, T2D. Through examination of literature, a broad range of themes was identified, pertaining to analysis performed on consumer-generated sources either independently or combined with another source. Most of the studies used ML algorithms to perform their analysis, speaking to the complexity of these data sets. These algorithms included k-means clustering, neural networks, decision trees, SVMs, and RF. PGHD is well suited for behavior insights in that the data can be collected far more frequently and they provide greater context than the coarse observations obtained during clinic visits and from static laboratory results.

The most prevalent theme from this review suggests that analysis of PGHD has the potential to detect undiagnosed diabetes or obesity or predict risk of developing diabetes or obesity [16-25]. Detection of T2D in the early stages or before onset can inform users of their risks and allow them to make necessary behavior changes to mitigate the risk of progression of the disease or further complications. Furthermore, models built to assess risk and manage T2D can be applied to other chronic diseases [56]. However, from our review, predicting risk requires structured data from questionnaires or surveys.

In addition to predicting the likelihood of disease, PGHD from people living with diabetes could also predict glycemic events [21,26-28,30,31]. However, this type of prediction requires commercial BGMs or CGMs in which the data are entered into an app either manually or automatically. People living with T2D are encouraged to frequently self-monitor blood glucose levels to obtain feedback on the healthy behavior changes already made, allowing treatments to be adjusted if necessary [61].** **Being able to predict glycemic events adds further feedback about treatment regimens in relation to glycemic control, allowing patients and providers to adjust care plans accordingly.

Another significant theme discovered through this review was the identification of factors that are characteristic of diabetes and obesity through PGHD from sources that include a combination of questionnaires or surveys, social media activity, and activity sensors [21,23,25,35-39]. It was specifically found through this review that diet and sleep quality were health behaviors that were contributing factors with regard to diabetes and obesity [23,26,37,38]. Furthermore, analysis of large data sets through PGHD was able to detect patterns or clusters of different health behaviors that are characteristic of people living with diabetes; the sources included surveys and apps [28,31,50]. Finally, PGHD regarding health behaviors can provide insights into new clinical findings related to diabetes [32-34]. From our review, these insights were obtained by analyzing passive activity collected from devices. Analysis of PGHD can provide new insights into the disease and the behaviors of people living with it, and further research may benefit by examining the intersectionality of behavioral clusters and patient outcomes.

Activity sensors served as another data source, passively collecting data about physical activity and sedentary behaviors. Whereas exercise is an important treatment for managing T2D, sedentary behaviors while awake are associated with premature mortality, increased BMI, increased glycated hemoglobin levels, increased adiposity, and hyperglycemia [57]. Even with regular moderate to vigorous physical activity, adverse health outcomes are associated with prolonged periods of sedentary time, which should be considered a separate behavior from physical activity, and people living with T2D should follow some exercise regimen while reducing time spent on sedentary behaviors [62]**.** Through this review, it was found that PGHD from activity sensors could detect patterns of sitting behaviors and find increased insulin resistance and higher triglyceride concentrations with increased sitting behaviors [28,44-48]. Further research may be necessary to examine the correlation of physical activity with sedentary behaviors in relation to the glycemic outcomes of people living with T2D.

Social media provided another data source to examine how people living with T2D manage their condition [35,37,39-41]. Social media sites not only provide a platform for people living with T2D to discuss their disease and share information, but also provide epidemiological insights into geographical correlations of the disease [35,41] as well as microinsights into the emotional status of people living with obesity or diabetes [39,42]. Further research may be necessary to examine a longitudinal picture of disease progression through social media analysis.

As found by Kitsiou et al [6] and Wang et al [7], mHealth interventions show promise for improving outcomes and increasing treatment adherence for people living with T2D. Our review expands on these findings by measuring adherence and outcome through analytics from PGHD [28,51-54]. Although prescriptive interventions that personalize messages have been shown to increase adherence [51,53], digital tracking tools were also shown to have increased adherence to treatment as opposed to standard treatment through logbooks [54]. These findings suggest that PGHD can provide granular insights into adherence to treatments and assess which treatment interventions are likely to increase adherence. Further research may be necessary to examine how certain interventions correlate to adherence.

Diabetes management and fitness technologies, as well as the use of social media, clearly constitute a rich data set for behavioral insights. Their frequent use, and in some cases continuous acquisition of detailed, relevant, and contextual data, gives unprecedented ability to develop applications for prediction, prognosis, and self-management insight. This provides a glimpse of the potential of using PGHD for other chronic conditions that lend themselves to similar frequent acquisition of quantitative data, such as cardiac conditions. Other conditions such as respiratory disease, mental health, and chronic pain are more elusive in this respect because of their qualitative mode of characterizing the condition. Future innovations should look to the advances made in diabetes management with respect to PGHD to consider these conditions.

### Limitations

This detailed review was conducted by only 1 reviewer, with a second reviewer contributing to the selection of studies and the remaining authors providing revision suggestions and commentary to the final draft of the paper. As all these studies contained PGHD, the accuracy of the data is limited to what was reported by the patients or what was passively collected through external devices. Furthermore, because some studies contained PGHD sources combined with other sources, some of the findings are not completely representative of PGHD. Moreover, the studies may not necessarily consider real-life use patterns because they may have been conducted in controlled settings. In addition, the scope of this study could not determine the efficacy of these approaches when implemented practically. Finally, the inclusion and exclusion criteria were developed by the authors based on their best knowledge of the subject, and articles were selected if the 2 reviewers (MSN and AB) believed that the articles met these criteria. Quality was assessed against the authors’ current knowledge of the topic, and they excluded articles if they believed that the articles contained information that contradicted their current knowledge.

### Conclusions

The emergence of technology-enabled tools that support individuals to manage their diabetes has resulted in the creation of a repository of PGHD to use ML algorithms to gather analytical insights into the health behaviors of people living with T2D, which otherwise cannot be gathered through other data sources. This review identified that analytics from PGHD have the potential to predict disease and outcomes, identify factors contributing to disease, investigate behavior patterns, discover new clinical findings, and improve adherence to treatments. Further research may benefit from examining the intersectionality of these concepts to create cohesive treatment plans for managing T2D.

## Appendix

Multimedia Appendix 1 Charting of scoping review studies.

## Abbreviations

AI: artificial intelligence
BGM: blood glucose meter
CGM: continuous glucose monitor
EMR: electronic medical record
mHealth: mobile health
ML: machine learning
PGHD: patient-generated health data
RF: random forest
SVM: support vector machine
T2D: type 2 diabetes
