# Supplementary material for: Patient-Generated Data Analytics of Health Behaviors of People Living With Type 2 Diabetes: Scoping Review
Source: JMIR Diabetes. 2021 Dec 20;6(4):e29027. doi: 10.2196/29027 (PMC8726031; doi:10.2196/29027)
Supplement: Multimedia Appendix 1 [file diabetes_v6i4e29027_app1.docx]

# Appendix – Data Extraction from Studies

Table 3 Charting of Scoping Review Studies

| Author | Title | Study Goals | Data Sources | Study Type | Analytical Method | Analytics Type | Main Findings |
| --- | --- | --- | --- | --- | --- | --- | --- |
| Namayanja J, Janeja V (2012) | An assessment of patient behavior over time-periods: a case study of managing type 2 diabetes through blood glucose readings and insulin doses | "Our goal is to understand if there is any trend in the patterns of the blood glucose measurements and the doses of regular insulin." | UCI Diabetes Dataset | Cross sectional study | K-means clustering | Descriptive | Clustering was able to detect granular behavioural patterns correlating to blood glucose levels and insulin dosages more accurately than statistical analysis. This study was able to determine which time periods were care needs more imminent amongst patients managing T2D. |
| S. Fong, Y. Zhang, J. Fiaidhi, O. Mohammed, S. Mohammed (2013) | Evaluation of stream mining classifiers for real-time clinical decision support system: a case study of blood glucose prediction in diabetes therapy | "The objective of this paper is twofold. We want to find out the most suitable classifier for [real-time clinical decision support system], and therefore we compared them in a diabetes therapy scenario. Also, we want to test the performance of the classifier candidate all-rounded with a real-time case study, as a preliminary step to validate the efficacy of the rt-CDSS as a whole." | Empirical Dataset from AAAI Spring Symposium  UCI Diabetes Dataset | Comparative Study | Very Fast Decision Tree (VFDT) iOVFDT Artificial Neural Network (ANN) Bayes | Predictive | A rt-CDSS must be able to 1) handle live streams, 2) have short time delay, 3) have accurate and consistent performance. This study found that ANN gave unsatisfactory performance, Bayes had the highest consistency but some outliers, VFDT had the highest accuracy with the study dataset but may not for all, and iOVFDT had the most stable performance. This classifier may be applicable to other clinical areas beyond diabetes. |
| X.H. Meng, Y.X. Huang, D.P. Rao, Q. Zhang, Q. Liu (2013) | Comparison of three data mining models for predicting diabetes or prediabetes by risk factors | "The purpose of this study was to compare multiple prediction models for diabetes incidence based on common risk factors." | Questionnaire (demographics, characteristics, family diabetes history, anthropometric measurements, lifestyle risk factors) | Comparative Study | Linear Regression ANN  Decision Tree (C5.0) | Predictive | ANN gave the lowest classification accuracy of 73.23% with a sensitivity of 82.18% and specificity of 64.49%; Logical regression achieved a classification accuracy of 76.13% with a sensitivity of 79.59% and a specificity of 72.74%; C5.0 achieved the best classification accuracy 77.87% with a sensitivity of 80.68% and specificity of 75.13%. |
| Hidalgo J, Maqueda E, Risco-Martín J, Cuesta-Infante A, Colmenar J, Nobel J (2014) | glUCModel: A monitoring and modeling system for chronic diseases applied to diabetes | Create a web application for patients and providers to communicate, have e-learning modules, make recommendations, and predict glucose levels | Web Application: Blood glucose Food Exercise Medications  Lab Results  Glucometers | Analytical Framework / Prototype | Case based reasoning for prescribing educational topics and lifestyle changes based on uploaded data  Grammatical Evolution for modelling glycemia | Descriptive / Predictive / Prescriptive | This framework was developed and would be evaluated for use in future studies. Model successfully predicted hypoglycemia between 79 - 100% in 6 patients, and successfully predicted hyperglycemia between 74 - 97%. |
| S.B. Choi, W.J. Kim, T.K. Yoo, J.S. Park, J.W. Chung, Y.H. Lee, et al. (2014) | Screening for prediabetes using machine learning models | "This study aimed at developing an intelligence-based screening model for prediabetes." | Korea National Health and Nutrition Examination Survey 2010 | Comparative Study | ANN & Support Vector Machines (SVM) to predict likelihood of prediabetes  Logistic regression to test accuracy of the model | Predictive | The SVM model had a higher accuracy in predicting prediabetes with an area under curve (AUC) of 0.731 compared to ANN that had an AUC of 0.729. |
| Li, Huan; Zhang, Qi; Lu, Kejie (2015) | Integrating Mobile Sensing and Social Network for Personalized Health-Care Application | "The main idea is to use smartphone to unobtrusively record and analyze the user’s physical activity and health status, and at the same time obtain the personalized health food recommendations from the remote server." | Smartphone input  Sensor data  Knowledge of life sciences  Social media  Restaurant Databases | Analytical Framework / Algorithm | Classification algorithms (J48, KNN, END, Bagging, Regression, Rotation Forest) to classify activity  Collaborative Filtering Model (CF) for personalizing recommendations | Prescriptive | Rotating Forest most accurately recognized physical activity with 73% accuracy. Overall, users were satisfied with recommendations made by system. |
| Abbar, Sofiane; Mejova, Yelena; Weber, Ingmar (2015) | You Tweet What You Eat: Studying Food Consumption Through Twitter | "In this work we examine the potential of Twitter to provide insight into US-wide dietary choices by linking the tweeted dining experiences of 210K users to their interests, demographics, and social networks." | Twitter  US Census Data | Cross sectional study | Longest n-gram matching to detect foods in Tweets  Pearson & Spearman correlations of "tweet" caloric values and state diabetes & obesity rates | Descriptive | Foods mentioned in tweets are predictive of obesity (r = 0.77) & diabetes (r = 0.66) statistics across the US states. |
| L. Han, S. Luo, J. Yu, L. Pan, and S. Chen (2015) | Rule extraction from support vector machines using ensemble learning approach: An application for diagnosis of diabetes | Screening for diabetes from the China Health & Nutrition Survey through rule extraction | China Health & Nutrition Survey | Comparative Study | SVM Classifier  C4.5, Naïve Bayes Tree, RF, CP Neural Networks for comparison  Then, SV + RF & SV + C4.5 for rule extraction | Predictive | The SVM + RF model yielded 89.6% precision and 44.3% recall, suggesting that that SVM combined with RF is superior to RF alone. |
| Fioravanti A, Fico G, Salvi D, García-Betances RI, Arredondo M (2015) | Automatic messaging for improving patient’s engagement in diabetes management: an exploratory study | "Our research seeks to determine whether a message sent in a proper time and with proper content has an impact on patients’ adherence to therapy" | Clinical data  Patient inputted data on mobile app | Randomized Control Trial (RCT) | Mathematical Formulae for estimating usage & adherence | Prescriptive | Prescriptive interventions that were personalized lead to higher usage and adherence to prescriptions during the 4-week study. |
| Chen, Yu; Randriambelonoro, Mirana; Geissbuhler, Antoine; Pu, Pearl (2016) | Social Incentives in Pervasive Fitness Apps for Obese and Diabetic Patients | "We investigate the effectiveness of social incentives for obese and diabetic patients using HealthyTogether as an experimental platform." | Healthy Together Android app (dyads of participants)  Fitbit sensors | Cohort Study | Linear Mixed Method | Descriptive | Compared to baseline, after connecting with a buddy, participants improved their daily floors from 7.72 floors / day to 9.17 floors / day. Participants improved on steps form 6332 steps / day to 6631 steps / day. Weak ties, however, proved to be demotivating. |
| Q. He; E. O. Agu (2016) | Towards sedentary lifestyle prevention: An autoregressive model for predicting sedentary behaviors | "In this work, our goal is to predict sedentary behavior based on user’s objective historical activity data rather than subjective terms, which are hard to evaluate and often inaccurate" | Public "StudentLife Dataset" that collected students' smartphone activity and fitness tracking through the accelerometer | Cross sectional study | Autoregressive (AR) Model Maximum Entropy Method (MEM) | Descriptive / Predictive | Model detected patterns of sedentary behaviours and demonstrated that people's sedentary behaviours are strongly correlated with historic sedentary behaviours in the past 6-hour windows, and patterns are repeated daily and weekly. |
| S. Tirunagari; S. Bull; S. Kouchaki; D. Cooke; N. Poh (2016) | Visualisation of survey responses using self-organising maps: A case study on diabetes self-care factors | "In this study, we use self-organising maps (SOMs) to visualise the responses of patients who share similar responses to survey questions, with the goal of helping clinicians understand how patients are managing their treatment and where action should be taken" | Surveys: Blood glucose Lifestyle Insulin  Administrative Data  Demographics | Cross sectional study | Self Organizing Maps (SOM) | Descriptive | Visualization detected clusters in which patients who took correct dosages of insulin had a tendency to take their injections at the correct time; patients who ate on time had a tendency to manage their food portions; and patients who checked their blood glucose with a monitor had a tendency to adjust their insulin dosage and carry snacks. |
| Abdullah, F. S., Manan, N. S. A., Ahmad, A., Wafa, S. W., Shahril, M. R., Zulaily, N., ... & Ahmed, A (2016) | Data Mining Techniques for Classification of Childhood Obesity Among Year 6 School Children | "The aim of this study was to identify the factors that influence the childhood obesity using various feature selection techniques. Other than that, this paper reports the application of various classifiers for the classification of childhood obesity." | National Physical Fitness Standard for Malaysian School Children Questionnaire | Comparative Study | Feature Selection Techniques: CfsSubsetEvaluator Consistency  Search Methods: Best first Genetic Search Greedy Stepwise Linear Forward  Classifiers: BayesNet Naïve Bayes Decision Tree (J48) Neural Network Multi-layer Perceptron SMO | Predictive | Classifiers were tested against various feature selection groups and search methods with J48 and SMO being the best classifiers to predict childhood obesity. CfsSubsetEvaluator with genetic search improved accuracy of classifiers. |
| Xiao, Houping; Gao, Jing; Vu, Long; Turaga, Deepak S (2017). | Learning Temporal State of Diabetes Patients via Combining Behavioral and Demographic Data | "In this paper, we propose a novel framework to capture the trajectory of latent states for patients from behavioral data while exploiting their demographic differences and similarities to other patients" | Demographic data  Sensor data | Analytical Framework / Algorithm | Gaussian distribution to validate each behavioural feature  Hypothesis test to validate demographic data  Demographic feature Hidden Markov Model (DfHMM) to estimate trajectory of latent states  Markov Chain Monte Carlo techniques to estimate model parameters | Predictive | DfHMM model proven to be effective at predicting the trajectory of latent states for each patient based on experiments with both synthetic and sensor data. |
| Gu, Weixi; Zhou, Yuxun; Zhou, Zimu; Liu, Xi; Zou, Han; Zhang, Pei; Spanos, Costas J.; Zhang, Lin (2017) | SugarMate: Non-Intrusive Blood Glucose Monitoring with Smartphones | "We propose Md3RNN, an efficient learning paradigm to make full use of the available blood glucose information. Specifically, the newly designed grouped input layers, together with the adoption of a deep RNN model, offer an opportunity to build blood glucose models for the general public based on limited personal measurements from single-user and grouped-users perspectives" | SugarMate App & sensors: Food intake Drug intake Insulin intake Physical activity Sleep quality | Mixed methods | Deep recurrent neural network (RNN) | Predictive | SugarMate was able to predict blood glucose levels exercise and sleep quality with manual records of food, drug, and insulin intake. Tested on a dataset of non-diabetics and people living T1D or T2D for 6 days, it presented an accuracy rate of 82.14%. |
| Al-Ramahi, Mohammad A.; Liu, Jun; El-Gayar, Omar F. (2017) | Discovering Design Principles for Health Behavioral Change Support Systems: A Text Mining Approach | "Using mobile diabetes applications as an example of Health [Behavioural Change Support Systems], we use topic modeling to discover design principles from online user reviews. We demonstrate the importance of the design principles through analyzing their existence in users’ complaints." | Online reviews of diabetes apps on the Apple iTunes store | Cross sectional study | Topic Modelling - Latent Dirichlet Allocation (LDA) | Descriptive | By extracting the text of online reviews of diabetes applications on the iTunes store and using topic modelling, the most important design principles were "effort expectancy", "self-monitoring", "informative presentation", "communication with doctors", and "integration with information systems". Other critical design principles are "integration with medical devices", "customization", and "technical support". The least important principles were "social support" and "persuasive messages". Users bothered most by issues pertaining to "technical support", "integration with information systems", "integration with medical devices", "effort expectancy", "customization", and "communication with doctors". |
| Nag, Nitish; Pandey, Vaibhav; Jain, Ramesh (2017) | Live Personalized Nutrition Recommendation Engine | "Our recommendation engine is driven by the primary goal of lowering the barriers to a personalized healthy choice when eating out, by distilling dish suggestions to a single contextually aware and easily understood score" | Restaurant database  Nutrition database  Sensor data  Self reported data | Analytical Framework / Algorithm | Adaptive Daily Value algorithm to estimate user's nutritional requirements  ELIXIR - ranked meals based on user's health needs | Prescriptive | Prototype created and tested on users. ELIXIR algorithm had highest correlation to dietitian from a previous study and its selections were validated by a dietitian in this study. |
| Q. He; E. O. Agu (2017) | A Rhythm Analysis-Based Model to Predict Sedentary Behaviors | "In this paper, we propose a lightweight model to predict future sedentary behaviors, facilitating prevention rather than reactive interventions. | Wearables such as Fitbit from Dartmouth StudentLife Dataset | Cross sectional study | AR Model  History-dependent model  History-free model  Hybrid model that combines sedentary history with circadian rhythms | Predictive | Cyclical rhythms of sedentary behaviours were more common than linear rhythms. |
| D. Machado; T. Paiva; I. Dutra; V. S. Costa; P. Brando (2017) | Managing diabetes: Pattern discovery and counselling supported by user data in a mobile platform | "The main objective of this work is to uncover usage patterns and to advise the user in these situations, through the use of the [advice ruled based system] and data-mining, guiding the user in general diabetic issues." | MyDiabetes mobile health | Analytical Framework / Algorithm | Advice Rule Based System (ARBS)  Association rules to reveal links, and weight of links, between variables  Bayesian networks for probablistic dependencies | Prescriptive | Framework developed where user manages glycemic values with alerts and education to avert crises. |
| Seixas AA, Henclewood DA, Langford AT, McFarlane SI, Zizi F, Jean-Louis G. (2017) | Differential and Combined Effects of Physical Activity Profiles and Prohealth Behaviors on Diabetes Prevalence among Blacks and Whites in the US Population: A Novel Bayesian Belief Network Machine Learning Analysis | "(1) estimate the prevalence of sedentary, moderately active, active, and very active lifestyles;  (2) determine the prevalence of diabetes across the different activity lifestyles;  (3) determine the prevalence of diabetes across different activity lifestyles between Blacks and Whites;  (4) simulate which physical activity profiles, as well as combination of behaviors and lifestyle factors (sleep, stress, and body mass index), among Blacks would yield a similar diabetes prevalence as Whites." | National Health Interview Survey | Cross sectional study | Bayesian Belief Network | Descriptive | This study determined prevalence of diabetes amongst black and white people with different health behavioural patterns. Amongst similar activity patterns, diabetes was more prevalent amongst blacks than whites. Physical activity combined with adequate sleep, low stress, and average body weight lowered diabetes prevalence amongst blacks. |
| Olivera AR, Roesler V, Iochpe C, Schmidt MI, Vigo A, Barreto SM, Duncan BB. (2017) | Comparison of machine-learning algorithms to build a predictive model for detecting undiagnosed diabetes - ELSA-Brasil: accuracy study. | "This paper presents the development and comparison of predictive models created from different machine-learning techniques with the aim of detecting undiagnosed type 2 diabetes, using baseline data from the Longitudinal Study of Adult Health (ELSA-Brasil)." | ELSA survey data | Comparative Study | Logistical regression ANN Naïve Bayes k-Nearest Neighbour Random Forest | Predictive | All algorithms produced an AUC of 70% or higher, but logistic regression was the most accurate with an AUC of 74%. |
| Albers DJ, Levine M, Gluckman B, Ginsberg H, Hripcsak G, Mamykina L (2017) | Personalized glucose forecasting for type 2 diabetes using data assimilation | "The goal of the research presented here is to generate personalized, accurate, and actionable predictions of glucose in response to nutrition that can assist individuals with diabetes in making quantitatively informed nutritional choices." | Data directly from participants (Meal images, Unstructured text descriptions, Glucose readings) | Analytical Framework / Algorithm | Data Assimilation using unscented Kalman filtering | Predictive | The findings from this model are as follows: 1) estimated data in real-time according to metrics, 2) forecasted in line with the opinions of certified diabetes educators, 3) personalized model to individual, 4) integrated with model selection machinery and chose the best model, 5) performed well given realistic data, 6) accurate output, 7) averaged in real time to produce accurate forecasts |
| Yom-Tov E, Feraru G, Kozdoba M, Mannor S, Tennenholtz M, Hochberg I. (2017) | Encouraging Physical Activity in Patients with Diabetes: Intervention Using a Reinforcement Learning System | "The aim of this study was to help type 2 diabetes patients increase the level of their physical activity." | Smartphone accelerometer | RCT | RL for personalizing messages | Predictive | Participants who received the personalized messages increased their physical activity and pace of walking compared to the control group. Moreover, participants experienced lower HbA1c levels, with greater reduction associated with increased adherence. RL algorithm improved in predicting messages to increase exercise. |
| Wilder, Bryan; Ou, Han Ching; de la Haye, Kayla; Tambe, Milind (2018) | Optimizing Network Structure for Preventative Health | "…we model a social influence processes where agents update their behavior (and thus weight status) in response to the behavior of those around them." | US Census  Los Angeles County Department of Public Health  LA Times Mapping LA Project  Facebook datasets | Analytical Framework / Algorithm | RECONNECT: Frank-Wolfe style algorithm  Greedy Dyad Greedy Groups (Linear Optimization) Random removals | Predictive | Initially, RECONNECT averted 230 cases of obesity. Algorithm outperforms baseline alternatives by 150%. |
| Heuschkel, Jens; Kauschke, Sebastian (2018) | More Data Matters: Improving CGM Prediction via Ubiquitous Data and Deep Learning | "In this paper we propose an approach to leverage activity information recorded by smartphones, data recordings of the patients’ insulin pump, continuous BG measurements, and heart rate data from a fitness tracker to improve their therapy decisions. Our solution provides the patients with predictive information to optimize their insulin injections, based on their past and current activities and BG level." | CGM  Insulin pumps  Smartphone recorded movements | Analytical Framework / Algorithm | Convolutional Neural Network (CNN) | Predictive | Model performed moderately better than existing commercial systems, with respect to mean squared error of the prediction, but still fails to detect critical situations. |
| S. Mitra; Y. Qiu; H. Moss; K. Li; S. L. Pallickara (2018) | Effective Integration of Geotagged, Ancillary Longitudinal Survey Datasets to Improve Adulthood Obesity Predictive Models | "Our goal is to predict an individual’s potential obesity down the line, given certain available information about him/her in the current day.... Our goal is to come up with a prediction model that can make better predictions of BMI by incorporating the growth chart data along with external factors from other available datasets." | National Longitudinal Survey of Youth 1997   US Census 2000 dataset  2000 CDC Growth Charts dataset | Analytical Framework / Algorithm | ANN  Gradient boosting  Random forest | Predictive | Compared to models that only use biometric attributes, this model improved accuracy in obesity prediction by empirically considering behavioural aspects (8.9% - 10.2%), environmental aspects (12.1% - 12.3%), and data uncertainty estimates (18.3% - 25.6%). |
| Q. Xue; X. Wang; S. Meehan; J. Kuang; J. A. Gao; M. C. Chuah (2018) | Recurrent Neural Networks Based Obesity Status Prediction Using Activity Data | "Thus, we develop a RNN based time-aware architecture to handle irregular observation times and identify relevant feature extractions from longitudinal patient records for obesity status improvement prediction." | Activity data from wearables  EMRs | Analytical Framework / Algorithm | RNN-based time aware architecture | Predictive | This model is able to capture underlying structures in users' time sequences with irregularities and achieve an accuracy of 77% to predicting obesity status. |
| Feller DJ, Burgermaster M, Levine ME, Smaldone A, Davidson PG, Albers DJ, Mamykina L.(2018) | A visual analytics approach for pattern-recognition in patient-generated data | "To develop and test a visual analytics tool to help clinicians identify systematic and clinically meaningful patterns in patient-generated data (PGD) while decreasing perceived information overload" | Meal photos  Pre- & post-meal blood glucose levels | Within Subject Design | Hierarchical clustering  Heatmap visualization | Descriptive | Registered Dietitians examined logbooks and Glucolyzer model to identify patterns of blood glucose levels and per-meal macronutrient composition. They found 50% more observations using Glucolyzer than logbooks, without losing any accuracy. |
| Weatherall J, Paprocki Y, Meyer TM, Kudel I, Witt EA. (2018) | Sleep Tracking and Exercise in Patients with Type 2 Diabetes Mellitus (Step-D): Pilot Study to Determine Correlations Between Fitbit Data and Patient-Reported Outcomes | "The aim of this study was to determine the direction and magnitude of associations between patient-generated health data (from the Fitbit Charge HR) and patient-reported outcomes for sleep patterns and physical activity in patients with type 2 diabetes mellitus (T2DM)." | Fitbit data (steps taken, minutes asleep)  Questionnaires | Cross sectional study | Descriptive statistics:  Continuous variables - means & standard deviations, t-tests to examine differences across groups  Categorical variables - percentages & frequencies, chi-square tests to examine differences between groups  Pearson correlations to measure associations between patient reported outcomes and Fitbit data | Descriptive | This study found a positive correlation between patient-generated data from Fitbit & patient reported outcomes to physical activity and sleep, with a stronger correlation with physical activity than with sleep. |
| A. Lincke, J. Lundberg, M. Thunander, M. Milrad, J. Lundberg, and I. Jusufi (2018) | Diabetes Information on Social Media | "The aim of our current efforts is to investigate the content and flow of information when people in Sweden use Twitter to talk about diabetes related issues." | Twitter | Cross sectional study | Word2Vector  k-means Clustering | Descriptive | This study found that 75% of Swedish diabetes tweets came directly from patients or family & friends to share diabetes related information and communicate their disease in an alternative way. |
| Hossain, R., Mahmud, S. H., Hossin, M. A., Noori, S. R. H., & Jahan, H. (2018) | PRMT: Predicting Risk Factor of Obesity among Middle-Aged People Using Data Mining Techniques | "This study aimed at developing a proposed data mining technique to predict obesity-based risk factor. We aimed to find human factors of obesity which are more important for human health...our research objectives are:  RO1: To examine the datasets by using data mining technique to predict the risk factor of obesity.  RO2: To examine the statistical data analysis to find which factor is more concern of obesity in Bangladesh." | Questionnaire | Cross sectional study / Comparative study | Statistical tools to predict major risk factors for obesity  WEKA to estimate accuracy and error measurement of data mining algorithms  Classifier: Naïve Bayes IBK Kstar Zeror Random tree Logistic | Descriptive / Predictive | This study suggests that 58% of people in their dataset are obese. Naïve Bayes was the most accurate classifier for predicting obesity. Age, heigh, weight, healthy lifestyle, marital status, BMI, economic, and sleep per a day were factors in predicting obesity class. |
| Ramazi, Ramin; Perndorfer, Christine; Soriano, Emily; Laurenceau, Jean-Philippe; Beheshti, Rahmatollah (2019) | Multi-Modal Predictive Models of Diabetes Progression | "Using this dataset, we created a model for predicting the levels of four major biomarkers related to T2D after a one-year period" | CGM  Activity monitoring sensors  Demographic data  Lab test results | Analytical Framework / Algorithm | Long Short-Term Memory RNN | Predictive | This study considers a large variety of factors which contribute to T2D. Predicting four biomarkers, there was a root mean square error of ±1.67% for HBA1c, ±6.22 mg/dl for HDL cholesterol, ±10.46 mg/dl for LDL cholesterol, and ±18.38 mg/dl for Triglyceride. |
| N. C. Pereira; J. D'souza; P. Rana; S. Solaskar (2019) | Obesity Related Disease Prediction from Healthcare Communities Using Machine Learning | "This work aims to overcome the above-mentioned limitations by developing a state-of-the-art system that streamlines machine learning algorithms for the effective prediction of Obesity and its related diseases considering the population of India" | Survey data  Input from medical professionals | Analytical Framework / Algorithm | Random Forest AdaBoost | Predictive | The model performed at "par excellence" with Random Forest and AdaBoost to predict obesity specifically to the Indian population. |
| Matsumoto, Kazuyuki; Ryu, Mopaa; Yoshida, Minoru; Kita, Kenji (2019) | Emotion Analysis on Weblog of Lifestyle Diseases | "In this paper, we investigate the relation between patient lifestyle and emotional state transition by analyzing weblog articles written by patients with diabetes." | Weblogs of Japanese diabetes patients | Cross sectional study | Feed Forward Neural Networks (FFNN) for text classifications  Bidirectional Encoder Representations from Transformer (BERT) and polarity vector score hybrid to classify sentences as positive or negative emotionally | Descriptive | When classifying keywords of webblogs, there were 90% recalls for categories "blood sugar level", "time", and "meal". However, "feeling", had a recall of less than 73.9%. Classifying the "feeling" sentences, the hybrid method classified emotional polarity with approximately 70% F1-score. There was a negative correlation between increased blood sugar and increase of positive emotion. |
| O. Oyebode; R. Orji (2019) | Detecting Factors Responsible for Diabetes Prevalence in Nigeria using Social Media and Machine Learning | "...our work aims to…[leverage] an African social media platform targeted at Nigerians to gather diabetes related data, and then applying machine learning technique to detect those factors responsible for diabetes prevalence in Nigeria." | Nairaland - African social network | Cross sectional study | Bag of Words model created through text  Naïve Bayes for text classification | Descriptive / Predictive | The model accuracy was 87.08%. Weight, food, and diet were found to be the most prevalent factors contributing to diabetes in Nigeria; while other less prevalent factors include pregnancy, age, and sleep. |
| Xie Z, Nikolayeva O, Luo J, Li D. (2019) | Building Risk Prediction Models for Type 2 Diabetes Using Machine Learning Techniques | "The goal of our study was to build predictive models for type 2 diabetes using 2014 BRFSS data by applying machine learning techniques, including support vector machine (SVM), decision tree, logistic regression, random forest, Gaussian Naive Bayes classifiers, and neural network. In addition, we expected to identify other risk factors for type diabetes using statistical methods." | 2014 Behavioural Risk Factor Surveillance System data (BRFSS) | Cross sectional study / Comparative study | SVM Decision Tree Logistic Regression Random Forest Gaussian Naïve Bayes classifiers Neural Networks | Predictive | All predictive algorithms had high AUCs ranging from 0.7182 to 0.7949. The neural network model had the highest accuracy (82.4%), specificity (90.2%), and AUC (0.7949), the decision tree model had the highest sensitivity (51.6%) for T2D. Sleeping 9 or more hours per day, or had a checkup frequency of less than 1 year had higher risk of developing T2D. |
| Faruqui SHA, Du Y, Meka R, Alaeddini A, Li C, Shirinkam S, Wang J. (2019) | Development of a Deep Learning Model for Dynamic Forecasting of Blood Glucose Level for Type 2 Diabetes Mellitus: Secondary Analysis of a Randomized Controlled Trial | "The objective of this work was to dynamically forecast daily glucose levels in patients with [T2D] based on their daily mobile health lifestyle data including diet, physical activity, weight, and glucose level from the day before." | Smartphone data:  Physical activity, weight, diet (LoseIt!)  Blood glucose levels (My GlucoHealth, DiabetesConnect App, Bluetooth-enabled meters - Entra Health Systems) | Cross sectional study | Long Short-Term Memory RNN | Predictive | Among 10 patients who were monitored daily for 6 months, this model was accurate in predicting next day glucose levels based on Clark Error Grid and ±10% range of the actual values. |
| Sarda A, Munuswamy S, Sarda S, Subramanian V. (2019) | Using Passive Smartphone Sensing for Improved Risk Stratification of Patients with Depression and Diabetes: Cross-Sectional Observational Study | "This study aimed to analyze the association between smartphone-sensing parameters and symptoms of depression and to explore an approach to risk-stratify people with diabetes." | Passive smartphone data (activity, mobility, sleep, communication)  Patient Health Questionnaire-9  Sociodemographic data | Cross sectional study / Comparative study | Descriptive analysis - representation of participants based on sociodemographic factors, clinical presentation, and mental well-being  Univariate analysis - differences between participants who show symptoms of depression and those who did not  Classification Modelling Comparison: SVM Decision Tree Random Forest Adaptive Boosting Extreme Gradient Boosting | Descriptive | Participants who displayed symptoms of depression had lower activity levels and lower social contacts than those who did not. The Extreme Gradient Boosting algorithm proved to have the best performance with a cross validation accuracy of 79.07% and test accuracy of 81.05% to classify symptoms of depression. |
| W. N. Ismail; M. M. Hassan; H. A. Alsalamah; G. Fortino (2020) [63] | CNN-Based Health Model for Regular Health Factors Analysis in Internet-of-Medical Things Environment | "...we proposed a new model for the discovery of regular correlated health-related factors to detect any abnormality in health status. This model can extract all the available regular factor behavior to create the implicit knowledge related to remote monitoring and managing human lifestyle." | Korea National Health and Nutrition Examination data  EMR data | Analytical Framework / Algorithm | CNN-regular target detection  Recognition model based on the Pearson Correlation Coefficient | Descriptive | The model uses multivariate analysis to select significant health factors, then it classifies these health factors in the second layer. It provides knowledge of correlated health parameters of obesity, hypertension, and diabetes. |
| Chang YJ, Bellettiere J, Godbole S, Keshavarz S, Maestas JP, Unkart JT, Ervin D, Allison MA, Rock CL, Patterson RE, Jankowska MM, Kerr J, Natarajan L, Sears DD. (2020) | Total Sitting Time and Sitting Pattern in Postmenopausal Women Differ by Hispanic Ethnicity and are Associated with Cardiometabolic Risk Biomarkers | "An objective of this study was to examine sedentary behavior and cardiometabolic health in Hispanic women versus nonHispanic women...We were also interested in evaluating whether associations of sedentary behavior and cardiometabolic biomarkers differed among Hispanic women and non-Hispanic women." | Accelerometer data | Cross sectional study | Random Forest to classify sitting behaviours  Multivariable linear regression to compare sitting among Hispanic and non-Hispanic women | Descriptive | Longer sitting time was associated with fasting insulin and triglyceride concentrations, insulin resistance, body mass index, and waist circumference amongst all women. These same outcomes were associated with longer mean sitting bout duration. The correlation between mean sitting bout duration and fasting glucose concentration was significantly stronger among Hispanic women than non-Hispanic women. |
| Zhang L, Shang X, Sreedharan S, Yan X, Liu J, Keel S, Wu J, Peng W, He M. (2020) | Predicting the Development of Type 2 Diabetes in a Large Australian Cohort Using Machine-Learning Techniques: Longitudinal Survey Study | "We aimed to develop a substantially improved diabetes risk prediction model using sophisticated machine-learning algorithms based on a large retrospective population cohort of over 230,000 people who were enrolled in the study during 2006-2017." | Baseline questionnaire with socioeconomic, health, and lifestyle factors | Longitudinal / Comparative | Conventional Logistical regression model  For comparison: Random Forest ANN Gradient Boosting | Predictive | Machine learning algorithms performed better than conventional regression analysis to predict diabetes risk. The Gradient Boosting algorithm had the most superior performance with an AUC of 79% three-year prediction and 75% in 10-year prediction. All ML models predicted BMI as the most significant contributing factor to diabetes. Reducing BMI to a healthy range would reduce the 10-year probability of diabetes onset 8.3% to 2.8%. |
| Liu Y, Yin Z. (2020) | Understanding Weight Loss via Online Discussions: Content Analysis of Reddit Posts Using Topic Modeling and Word Clustering Techniques | "This study aimed to investigate the extent to which the content posted by users in the r/loseit subreddit, an online community for discussing weight loss, and online interactions were associated with their weight loss in terms of the number of replies and votes that these users received." | Reddit posts on r/loseit | Cross sectional study | Topic Modelling & Hierarchical Clustering for global topics and word semantic clusters  Regression Analysis to analyze relationship between weight loss and topics, word semantic clusters, online interactions | Descriptive | By analyzing the content of Reddit posts, start weight, active days, median number of votes, mentions of exercise, and nutrition were associated with higher weight loss. Those who lost more weight may have been motivated by negative emotions. Mentions of vacations, payments, employment status, and family members was associated with less weight loss. |
| Griffis H, Asch DA, Schwartz HA, Ungar L, Buttenheim AM, Barg FK, Mitra N, Merchant RM. (2020) | Using Social Media to Track Geographic Variability in Language About Diabetes: Analysis of Diabetes-Related Tweets Across the United States | "This study aimed to characterize the language of Twitter users’ posts regarding diabetes and describe the correlation of themes with the county-level prevalence of diabetes." | Twitter Data | Cross sectional study | LDA | Descriptive | Themes from tweets that contained the term diabetes include unhealthy food and drink, treatments, symptoms / diagnosis, research, risk factors, recipes, news, healthcare, management, fundraising, diet, communications, supplements / remedies. Themes of unhealthy foods are most correlated with geographical areas with higher prevalence of diabetes and themes of research were negatively correlated with disease prevalence. Topics varied by geography. |
